# Early circulating tumor DNA changes predict outcomes in head and neck cancer patients under re‐radiotherapy

**DOI:** 10.1002/ijc.35152

**Published:** 2024-08-30

**Authors:** Florian Janke, Florian Stritzke, Katharina Dvornikovich, Henrik Franke, Arlou Kristina Angeles, Anja Lisa Riediger, Simon Ogrodnik, Sabrina Gerhardt, Sebastian Regnery, Philipp Schröter, Lukas Bauer, Katharina Weusthof, Magdalena Görtz, Semi Harrabi, Klaus Herfarth, Christian Neelsen, Daniel Paech, Heinz‐Peter Schlemmer, Amir Abdollahi, Sebastian Adeberg, Jürgen Debus, Holger Sültmann, Thomas Held

**Affiliations:** ^1^ Division of Cancer Genome Research, German Cancer Research Center (DKFZ) Heidelberg Germany; ^2^ German Center for Lung Research (DZL), TLRC Heidelberg Heidelberg Germany; ^3^ National Center for Tumor Diseases (NCT) Heidelberg Germany; ^4^ Department of Radiation Oncology Heidelberg University Hospital Heidelberg Germany; ^5^ Heidelberg Institute of Radiation Oncology (HIRO) Heidelberg Germany; ^6^ Clinical Cooperation Unit Radiation Oncology, German Cancer Research Center (DKFZ) Heidelberg Germany; ^7^ Junior Clinical Cooperation Unit, Multiparametric Methods for Early Detection of Prostate Cancer, German Cancer Research Center (DKFZ) Heidelberg Germany; ^8^ Department of Urology University Hospital Heidelberg Heidelberg Germany; ^9^ Faculty of Biosciences Heidelberg University Heidelberg Germany; ^10^ Heidelberg Ion Beam Therapy Center (HIT) Heidelberg Germany; ^11^ German Cancer Consortium (DKTK) Heidelberg Germany; ^12^ Division of Radiology, German Cancer Research Center (DKFZ) Heidelberg Germany; ^13^ Department of Neuroradiology Bonn University Hospital Bonn Germany; ^14^ Department of Radiotherapy and Radiation Oncology Marburg University Hospital Marburg Germany; ^15^ Marburg Ion‐Beam Therapy Center (MIT), Department of Radiotherapy and Radiation Oncology Marburg University Hospital Marburg Germany; ^16^ Universitäres Centrum für Tumorerkrankungen (UCT) Frankfurt Marburg Germany

**Keywords:** circulating tumor DNA, copy number variations, head and neck cancer, predictive biomarker, re‐radiotherapy

## Abstract

Local recurrence after radiotherapy is common in locally advanced head and neck cancer (HNC) patients. Re‐irradiation can improve local disease control, but disease progression remains frequent. Hence, predictive biomarkers are needed to adapt treatment intensity to the patient's individual risk. We quantified circulating tumor DNA (ctDNA) in sequential plasma samples and correlated ctDNA levels with disease outcome. Ninety four longitudinal plasma samples from 16 locally advanced HNC patients and 57 healthy donors were collected at re‐radiotherapy baseline, after 5 and 10 radiation fractions, at irradiation end, and at routine follow‐up visits. Plasma DNA was subjected to low coverage whole genome sequencing for copy number variation (CNV) profiling to quantify ctDNA burden. CNV‐based ctDNA burden was detected in 8/16 patients and 25/94 plasma samples. Ten additional ctDNA‐positive samples were identified by tracking patient‐specific CNVs found in earlier sequential plasma samples. ctDNA‐positivity after 5 and 10 radiation fractions (both: log‐rank, *p* = .050) as well as at the end of irradiation correlated with short progression‐free survival (log‐rank, *p* = .006). Moreover, a pronounced decrease of ctDNA toward re‐radiotherapy termination was associated with worse treatment outcome (log‐rank, *p* = .005). Dynamic ctDNA tracking in serial plasma beyond re‐radiotherapy reflected treatment response and imminent disease progression. In five patients, molecular progression was detected prior to tumor progression based on clinical imaging. Our findings emphasize that quantifying ctDNA during re‐radiotherapy may contribute to disease monitoring and personalization of adjuvant treatment, follow‐up intervals, and dose prescription.

## Graphical abstract

What's new?

Head and neck cancer frequently returns after radiation therapy, and re‐irradiation is commonly used to control locally advanced disease. Here, the authors analyzed circulating tumor DNA (ctDNA) as a predictive marker of disease progression after initial radiation treatment. They tested plasma samples from 16 patients at four timepoints during re‐irradiation and at follow‐up visits. In five cases, molecular progression was detected before tumor progression based on imaging. Detection of ctDNA in plasma samples during and after re‐irradiation correlated with short progression‐free survival. Quantitative ctDNA analysis may therefore be an effective way to monitor disease progression and personalize follow‐up treatment.

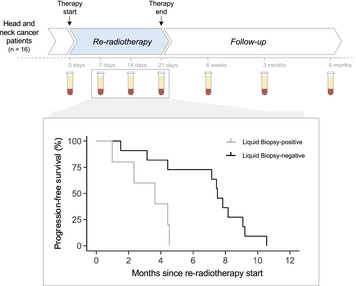

## 1 BACKGROUND

Local recurrences after definitive radiotherapy (RT) are frequent in locally advanced head and neck cancer (HNC) patients. As the majority of these patients are not amenable to complete resection, alternative therapeutic options are needed for adequate disease management.^1^, ^2^, ^3^ Re‐irradiation (re‐RT), preferably with advanced radiation techniques, is routinely considered in patients with locally and/or regionally recurrent or persistent head and neck cancer in the absence of distant metastatic disease. Re‐RT (alone or in combination with chemotherapy) was demonstrated to improve survival compared to chemotherapy, but was frequently accompanied by significant treatment‐related adverse events.^1^, ^2^, ^4^ Intensity modulated radiation therapy is widely used for re‐RT of locally recurrent HNCs and novel radiation techniques—such as carbon ion radiation are under investigation due to their favorable toxicity profile and improved relative biological effectiveness.^5^, ^6^, ^7^ In general, the improved local control by application of higher total doses has to be weighted carefully against the increased risk of severe toxicity.^5^, ^8^ Despite the clinical benefits of re‐RT in recurrent HNC, overall survival (OS) remains dismal, and early tumor progression is common.^3^, ^9^ Hence, biomarkers predicting therapy success or disease progression are urgently needed to adjust therapy.

Liquid biopsies comprise the analysis of tumor‐derived molecules from peripheral blood samples of cancer patients. In particular, circulating tumor DNA (ctDNA) within patient plasma was demonstrated to provide minimally invasive access to cancer‐specific alterations such as mutations^10^, ^11^ or copy number variations (CNVs^12^, ^13^). The quantification of ctDNA in longitudinally collected plasma enables real‐time disease surveillance and identification of tumor progression prior to radiologic imaging.^14^, ^15^, ^16^, ^17^ Furthermore, growing evidence suggests that residual ctDNA after therapy can identify minimal residual disease and patients prone to early relapse.^18^, ^19^, ^20^, ^21^ In advanced HNC patients, cancer‐specific mutations in ctDNA were demonstrated to dynamically reflect therapy response and identified patients with short survival.^16^, ^22^, ^23^ Large‐scale CNVs are common in HNCs^24^ and, therefore, represent promising analytes for ctDNA‐based tumor evaluation. Plasma CNVs were rarely investigated in HNC patients^25^ and so far no study assessed cell‐free CNVs in the clinical setting of re‐irradiation of recurrent locally advanced HNC patients.

Here, we present the results of a prospective study correlating CNV‐based ctDNA dynamics with clinical outcome in locoregional recurrent HNC patients under re‐RT recruited within the “*Carbon Ion Re‐Radiotherapy in Patients with Recurrent or Progressive Locally Advanced Head‐and‐Neck Cancer*” (CARE^26^) phase II clinical trial.

## 2 METHODS AND MATERIALS

### 2.1 Patient selection

Ninety four longitudinal plasma specimens were collected from 16 patients with locally and/or regionally recurrent or persistent head and neck cancer scheduled for re‐radiotherapy. In some cases, re‐RT was scheduled following an incomplete resection. The patients were recruited as part of the CARE phase II randomized clinical trial (NCT04185974^26^). Plasma sample collection and re‐irradiation of HNC patients were carried out at the department of radiation oncology of the Heidelberg University Hospital. In addition, plasma specimens of 35 healthy donors (i.e., individuals without known current tumor disease) were collected at the urological department of the Heidelberg University Hospital and whole genome sequencing (WGS) data from plasma cell‐free DNA (cfDNA) of further 22 healthy donors was obtained from literature (Supplementary Table 1).^27^

### 2.2 Re‐radiotherapy

Patients were randomly assigned to carbon ion re‐RT (CIRT) or volumetric modulated arc therapy (VMAT) in accordance with established institutional standards and current NCCN guidelines recommending advanced radiation techniques. Patients were immobilized using a thermoplastic head‐mask system. For treatment planning, computed tomography (CT) scans with a slice thickness of 3 mm were utilized, and contrast‐enhanced T1‐weighted magnetic resonance imaging (MRI) was employed for image registration. The treatment planning process was performed using the planning software Syngo PT‐Planning (Siemens, Erlangen, Germany) including biologic plan optimization for carbon ion plans and Masterplan Oncentra MasterPlan® (Nucletron, Columbia, SC, USA), RayStation® (RaySearch Laboratories, Stockholm, Sweden) or Accuray Precision® Treatment Planning (Accuray, Sunnyvale, CA, USA) for photon plans. Only the visible tumor, apparent on contrast‐enhanced imaging, was delineated as the gross tumor volume (GTV). The total dose ranged between 51 and 60 Gy Relative Biological Effectiveness (RBE) of CIRT delivered in 6 fractions per week of 3 Gy (RBE) by controlled active raster scanning. BED2Gy represents the dose equivalent in conventional fractionation with 2 Gy fractions. The BED2Gy of CIRT was calculated from the physical dose using the local effect model (LEM1) and an alpha/beta value of 2,^7^ resulting in equivalent doses between 64 and 75 Gy of CIRT. The alpha/beta value was uniformly set at 2 to prevent severe treatment‐related toxicity, and the individual dose prescription was required to meet prespecified normal tissue constraints consistent with Quantec analyses.^28^ For photon re‐RT by VMAT, the total dose ranged between 54 to 60 Gy delivered in five fractions per week of 2 Gy. Image guidance was performed using orthogonal x‐rays and daily position correction or CT‐imaging and position correction, respectively. At progression following re‐RT, patients were treated as clinically indicated.

### 2.3 Assessment of treatment response and disease progression

Staging of (recurrent) disease was performed according to the AJCC/UICC‐TNM classification of malignant tumors, 8th edition. Therapy response and disease progression were determined according to the most recent Response Evaluation Criteria in Solid Tumors (RECIST) 1.1 criteria.^29^, ^30^ The initial assessment of the disease (including CT or MRI) was performed between surgical intervention and the beginning of re‐radiotherapy. Follow‐up assessments (including MRI or CT) were performed regularly until disease progression, or rejection by the patient, in accordance with NCCN and the institution's guidelines. (Local) Progression‐free survival (PFS) was measured as the time interval between the initiation of re‐irradiation and the date of (local) relapse/progression, or death due to any cause.

### 2.4 Blood sample processing and cfDNA isolation

Peripheral blood was collected by venipuncture and subjected to plasma isolation within 1 h. The timespan between blood collection and the application of radiotherapy is documented in Supplementary Table 2 (median = 16 min after re‐RT). Briefly, whole blood—collected in K_2_EDTA tubes—was centrifuged at room temperature for 15 min at 1500 ×*g* without brake. To exclude residual cell debris, the plasma was centrifuged again at 15°C and 15,000 ×*g* for 10 min and subsequently stored at −80°C until further use. cfDNA was isolated from 2 mL of clarified plasma using the QIAamp MinElute ccfDNA Kit (Qiagen, Hilden, Germany). DNA quantity and integrity was assessed by the Qubit dsDNA High Sensitivity Kit (Thermo Fisher Scientific, Waltham MA, USA) and the Fragment Analyzer 5200 system (Agilent Technologies, Santa Clara CA, USA), respectively.

### 2.5 Library preparation and low‐coverage WGS of cfDNA

Sequencing libraries for low‐coverage (lc)WGS were prepared as previously described.^31^ In brief, 1.2–5.0 ng of cfDNA were subjected to end‐repair, A‐tailing and sequencing adapter ligation using Roche's KAPA HyperPrep reagents with NEBNext Multiplex Oligos for Illumina adapters (New England Biolabs, Ipswich, USA). After a 15‐h adapter ligation period at 16°C, libraries were purified by a double‐sided size selection and amplified using 9–10 PCR cycles, following the manufacturer's instructions. In the following steps, libraries were pooled equimolarly and sequenced in 48 to 96‐plexes on a NovaSeq6000 instrument with S4 paired‐end 100‐bp flowcells. The lcWGS library preparation protocol of cfDNA samples obtained from literature were described in Peneder et al.^27^ Per sample library preparation statistics for all samples are given in Supplementary Table 2.

### 2.6 Sequencing data processing

Adapter sequences in raw fastq reads were trimmed using Cutadapt v3.7^32^ and mapped to the reference genome GRCh37/hg19 by BWA‐MEM v0.7.17^33^ with default settings. Subsequently, aligned reads were sorted by chromosomal coordinates and indexed using samtools v1.9.^34^ Picard's (v2.25.1) Markduplicates function was used to mark and collapse duplicate reads to allow one read per alignment position. The sequencing data quality was evaluated by fastqc v0.11.5 and mosdepth v0.3.8^35^ (Supplementary Table 2). Due to the higher sequencing depth of the data obtained from literature (Supplementary Table 2), we downsampled the corresponding bam files to approximate the average genome coverage of our in‐house HNC and healthy donor cohorts (i.e., 2.5×). Downsampling was performed by samtools v1.9.

### 2.7 Copy number variation profiling

Genome‐wide CNV profiles were determined by WisecondorX v1.2.5^36^ with default parameters, separating the genome into 1000‐kb bins. Fifty seven healthy donor cfDNA samples served as diploid references (panel‐of‐normals) for normalization. For the normalization of healthy donor samples, the panel‐of‐normals was reduced by the currently analyzed sample. Circular binary segmentation^37^ was utilized to define genomic sections of estimated equal copy numbers. To ensure the robustness of the obtained copy number profiles, we bootstrapped the panel‐of‐normals with 100 iterations, considering the most frequently called copy number state as the ‘true state’. Hereby, we confirmed that CNVs were not driven by individual reference samples within the panel‐of‐normals.

### 2.8 Determination of CNV‐based ctDNA burden

As part of the WisecondorX v1.2.5 framework, copy number profiles were summarized to a copy number abnormality (CPA) score which expresses the extent of chromosomal instability within a given cfDNA sample.^38^ To leverage the information available when CNVs are assessed in longitudinal samples, we devised a ctDNA‐informed CPA score (ctCPA). To this end, copy number states of the current and all previously collected samples of the same patient were combined into a cumulative, patient‐specific copy number profile, which encompassed copy number gains/losses detected in at least one of the serial samples. Segments with conflicting copy number states (i.e., gains and losses within the same region of two or more samples) were excluded (Supplementary Figure 1). ctCPA scores were calculated as shown in Equation (1).

(1) $$\text{ctCPA score}=\sum_{i=1}^{n}\left(Z_{{\text{segment}}_{i}}\times D_{{\text{segment}}_{i}}\right)/n$$

Here, n represents the number of copy number segments within a sample. The z‐score of a segment i is given by $Z_{{\text{segment}}_{i}}$ and $D_{{\text{segment}}_{i}}$ represents the directionality of a segment within the cumulative copy number profile and can take one of three values according to its copy number state (gain: +1; loss: −1; neutral: 0). Both CPA scores and ctCPA scores were calculated after exclusion of chromosome X/Y and represent the median across 100 panel‐of‐normal bootstrap iterations (as described in the previous section). (ct)CPA scores are expressed per 100‐Mb.

### 2.9 Statistical analyses and data visualization

Since CPA scores were not normally distributed as tested by the Shapiro–Wilk test, comparisons between independent and paired data were performed using the Mann–Whitney *U* and Wilcoxon's paired test, respectively. The Spearman rank correlation was used to relate GTV with CPA scores. Survival data were evaluated according to Kaplan–Meier and survival between groups was compared using the two‐sided log‐rank test. Univariate and multivariable survival analysis was carried out using Cox proportional hazard models. *p*‐Values <.05 were considered statistically significant. All statistical analyses were carried out using R v4.2.0 (R Foundation for Statistical Computing, Vienna, Austria) and relevant plots were generated with the ggplot2 R package.^39^

## 3 RESULTS

### 3.1 Patient characteristics

Ninety four longitudinal plasma samples from 16 locally advanced recurrent HNC patients were collected within the framework of the CARE clinical trial.^26^ The median age at the time point of study inclusion was 59 years (range 49–72 years) and the study population included 68.8% males. Fifty six percent of patients were current or former smokers and 14 out of 16 had a squamous cell carcinoma (Table 1 and Supplementary Table 1). Administered therapy regimens prior to study enrollment included radiotherapy (RT; 16/16), concomitant systemic therapy (9/16) and surgery (11/16). The median GTV at re‐irradiation baseline was 18.9 cm^3^ (range 0.5–139.1 cm^3^). Re‐RT regimens comprised CIRT (7/16) and VMAT (9/16). A median of 6 serial plasma samples were analyzed per patient. Blood plasma was collected at re‐RT baseline (16/16), after 5 (ca. +7 days; 16/16) and 10 fractions (ca. +14 days; 16/16) as well as at the end of re‐RT (ca. +21 days; 16/16). Additional samples were collected 6 weeks (14/16), three (7/16) and 6 months (9/16) after re‐RT termination (Supplementary Figure 2). Following re‐RT, 15 out of 16 patients received at least one line of salvage therapy (surgery, *n* = 6; chemotherapy, *n* = 2; immunotherapy, *n* = 4, immuno−/chemotherapy combination, *n* = 5). All patients demonstrated radiographic disease progression within the follow‐up period with a median time to progression of 5.8 months (range 1.0–10.6 months). First disease progression after re‐RT occurred at local (*n* = 10), regional (*n* = 2), local and regional (*n* = 1) or distant (lung metastasis *n* = 2, liver metastasis *n* = 1) sites. Six patients experienced a second progression (local *n* = 3, regional and regional *n* = 2, regional and distant *n* = 2, and distant *n* = 1) (Supplementary Table 1).

**TABLE 1:** Patient characteristics.

Head and neck cancer patients analyzed in this study (*n* = 16)		
Age, median (range)		59 (49–72)
Sex, %male		68.8%
Smoking status, %current or former smokers		56.3%
Histology, number of patients	Adenocarcinoma	2
	Squamous cell carcinoma	14
T stage, number of patients	<4	7
	4	9
N stage, number of patients	0	13
	≥1	3
M stage, number of patients	0	15
	1	1
Re‐irradiation modality, number of patients	CIRT	7
	VMAT	9
Localization of primary tumor, number of patients	Oral cavity	2
	Oropharynx	3
	Nasal cavity	3
	Nasopharynx	3
	Hypopharynx	2
	Sinuses	2
	Skull base	1
Histologic p16 expression in oropharyngeal carcinoma, number of patients	positive	1
	negative	2
EBV in nasopharyngeal carcinoma, number of patients	Positive	1
	Negative	1
	Unknown	1
%KPS, median (range)		80 (70–90)
GTV in cm^3^, median (range)		18.9 (0.5–139.1)
Serial plasma specimens per patient, median (range)		6 (4–7)
Progression‐free survival in months, median (range)		5.8 (1.0–10.6)
Overall survival in months, median (range)		15.5 (7.1–35.6)

Abbreviations: CIRT, carbon ion radiotherapy; EBV, Epstein–Barr virus; GTV, gross tumor volume; KPS, Karnofsky performance score; M, metastasis; N, node; T, tumor; VMAT, volumetric modulated arc therapy.

### 3.2 Copy number variation landscape in locally advanced HNC patients under re‐radiotherapy

**FIGURE 1:**
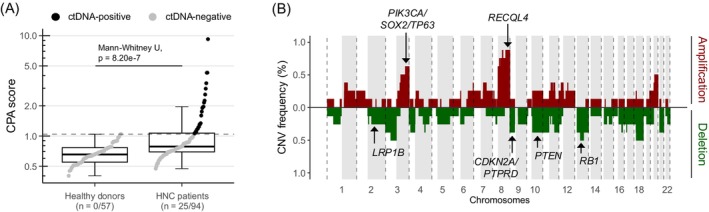
(A) Box plot comparing CPA scores between healthy donors and head and neck cancer (HNC) patients. The maximum CPA score across healthy donors served as ctDNA detectability threshold (dotted line; CPA = 1.044). ctDNA‐positive and ‐negative samples are colored black and gray, respectively. ctDNA‐positive and total sample number per group is given in brackets. Box plots represent median, upper and lower quartile with Tukey Whiskers. (B) Summary of recurrent CNVs in HNC patients with detectable ctDNA (*n* = 8). The y‐axis represents the frequency of a detected copy number state at the chromosomal coordinate given on the x‐axis. All longitudinal plasma samples were considered, however, recurrently detected CNVs in two or more samples of the same patient were only counted once. The CNV frequency (i.e., the number of occurrences in the eight patients assessed) of amplifications and deletions is given in red and green, respectively. Areas shaded in gray represent the q‐arm of the respective chromosome. Genes associated with HNC tumorigenesis are labeled.

HNCs are—like many other cancer types—characterized by complex CNV patterns across the genome.^24^ Here, we determined genome‐wide CNV profiles from cfDNA lcWGS data (median coverage = 2.8×, range 1.0×–15.1×; Supplementary Table 2) in our HNC cohort and 57 healthy donors as reference. For each cfDNA sample, the CNV profile was integrated into a metric (CPA score) to quantitatively assess the extent of chromosomal instability detectable in the circulation (Methods). The CPA score was used as a surrogate for ctDNA burden since cell‐free chromosomal instability metrics were demonstrated to relate to other ctDNA measures (e.g., mutations^13^, ^40^, ^41^, ^42^;) and tumor burden.^43^, ^44^ To define the ctDNA detection threshold, we determined CPA scores in the healthy individuals and set the maximum value as a cut‐off (median = 0.655, range 0.401–1.044). Using this threshold, we detected ctDNA in 25 out of 94 HNC patient samples (26.6%) and in 8 out of 16 patients (50.0%). HNC patient samples demonstrated a significantly higher CPA score compared to healthy individuals (Mann–Whitney *U*, *p* = 8.20e^⁻7^; Figure 1A). We also observed significantly elevated CPA scores between HNC patients and healthy donors when individual plasma collection time points were assessed (Supplementary Table 3).

The CNVs detected in the ctDNA‐positive patients resided in genomic regions known to HNCs. Recurrent copy number changes included amplifications of 3q, 8q, and 11q13/22 as well as deletions in 3p, 5q, 9p, and 13q (Figure 1B). These regions encompass tumor driver (e.g., *PIK3CA*) and suppressor genes (e.g., *CDKN2A*, *RB1*, and *PTEN*) recognized in HNCs as well as squamous lineage transcription factors (*TP63* and *SOX2*).^24^, ^45^

### 3.3 Large tumors prior to re‐RT were associated with increased ctDNA levels

Next, we evaluated the association between CPA scores and demographic as well as clinical parameters. No statistically significant CPA score difference was observed when stratifying patients by age, gender, disease dissemination (i.e., local vs. regional or distant metastasis) or smoking status. This was found for the combined analysis of all longitudinal plasma samples and when sampling time points were investigated on an individual basis (Supplementary Table 4). Furthermore, we observed no statistically significant difference in CPA scores when comparing patients treated with VMAT to CIRT therapy in the given cohort (Supplementary Figure 3 and Supplementary Table 4). The stratification of our HNC patients by the cohort's median GTV (18.9 cm^3^)—as determined at re‐RT baseline—revealed significantly higher CPA scores in larger tumors, considering all serial samples (Mann–Whitney *U*, *p* = 2.91e^⁻5^; Supplementary Figure 4A and Supplementary Table 4). The investigation of individual sampling time points showed significantly higher CPA scores in tumors ≥18.9 cm^3^ after 5 (*p* = .005) and 10 (*p* = .010) re‐RT fractions but not at baseline, re‐RT end and at follow‐up visits (Supplementary Figure 4B). Furthermore, we did not find a significant correlation between baseline GTV and CPA scores (Spearman, *⍴* = .050 and *p* = .856; Supplementary Figure 4C).

### 3.4 Integration of longitudinal plasma copy number profiles enhanced ctDNA detection

**FIGURE 2:**
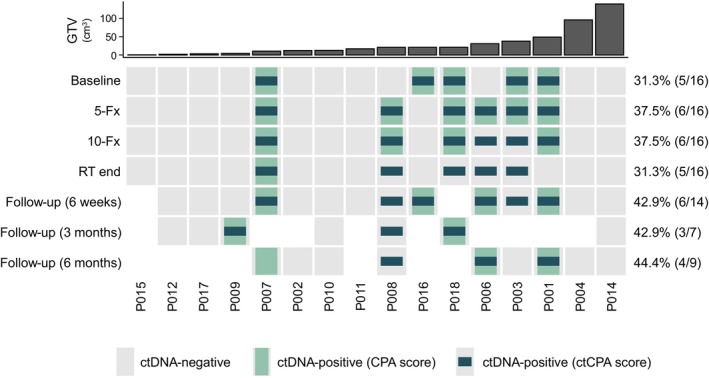
Head and neck cancer patient cohort overview highlighting the detectability of ctDNA copy number variation (CNV) analysis from low‐coverage whole genome sequencing. Green and blue colors indicate detectable CNVs by de novo CNV‐calling (CPA score) and using information from previous serial plasma samples of the same patient (ctCPA score), respectively. Missing squares represent unavailable plasma samples. The number of ctDNA‐positive and total number of samples per time point is given in brackets.

Previous reports showed that the focused analysis of patient‐specific alterations (e.g., mutations or CNVs) can enhance the sensitivity of ctDNA detection assays.^11^, ^46^ These studies utilized information from matched tumor tissue to direct their analysis to patient‐specific tumor alterations, thereby omitting background noise. Herein, we posited that a similar strategy could be employed in a disease monitoring scenario, using information from previous serial liquid biopsies to identify patient‐specific CNVs. To this end, we devised a ctDNA‐informed CPA score (ctCPA) that expresses the deviation from a copy number neutral state, focusing only on genomic regions with copy number changes identified in the current and all previous samples of the same patient (cumulative CNV profile; Methods and Supplementary Figure 1). As the cumulative CNV profiles were patient‐specific, thresholds denoting ctDNA‐positivity were established for each patient individually by determining the maximum ctCPA score at the given cumulative CNV profile across all 57 healthy donors. The ctCPA score detected ctDNA in 35 out of 94 samples (37.2%), increasing the ctDNA detectability by 10.6% compared to the CNV analysis without incorporation of longitudinal sample information (Figure 2). In total, 10 additional ctDNA‐positive samples in 4 patients were identified, while only one previously positive sample was classified as negative by the ctCPA score. The largest gain of samples with detectable ctDNA was observed at the time point of re‐RT termination. Here, ctDNA was found in 4 additional patients. The number of ctDNA‐positive patients remained unaltered (8/16) as a minimum of one ctDNA‐positive sample per patient is required for the employed serial CNV analysis.

### 3.5 Residual ctDNA during and immediately after re‐irradiation was associated with short progression‐free survival

**FIGURE 3:**
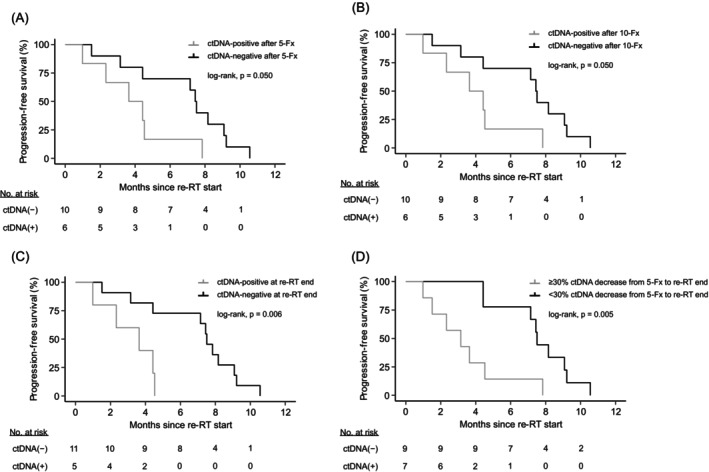
Association between ctDNA levels and progression‐free survival after re‐radiotherapy. Progression‐free survival (PFS) according to ctDNA detectability, as assessed via ctCPA scores, in plasma specimens collected after five fractions (A), 10 fractions (B) and at the end of re‐radiotherapy (re‐RT; C). (D) Association between PFS and CPA score change from samples taken after five re‐RT fractions to re‐RT end. Relative changes were calculated using CPA scores or ctCPA scores (if available). A decrease of ≥30% was used for partitioning of patients. Groups were compared using the two‐sided log‐rank test.

The prognostic and predictive value of ctDNA detection at therapy initiation and early post‐treatment time points has been reported in numerous studies.^18^, ^19^, ^20^, ^21^ Here, we evaluated whether quantifiable ctDNA levels (i.e., (ct)CPA scores exceeding the maximum scores of our healthy donor cohort) at re‐RT baseline, after 5 and 10 fractions of irradiation and at the end of re‐RT were associated with short time to tumor progression. ctCPA scores were calculated for the respective sampling time point (i.e., cumulative CNV profiles were only built up to the currently investigated time point), and ctDNA‐positivity was denoted based on the maximum ctCPA score in our healthy donors. We found no significant relation between ctDNA detectability and PFS at re‐RT baseline (log‐rank, *p* = .433; hazard ratio [HR] = 1.59; 95% confidence interval [CI] = 0.50–5.1; Supplementary Table 5). However, residual ctDNA after the administration 5 and 10 fractions of re‐RT (both: log‐rank, *p* = .050; HR = 3.04; 95% CI = 0.95–9.69; Figure 3A,B) and at therapy termination identified patient populations with short PFS (log‐rank, *p* = .006; HR = 6.18; 95% CI = 1.43–26.64; Figure 3C). Next, we evaluated whether detectable ctDNA can predict local disease control by re‐RT. We found no significant association between ctDNA detectability and time to local disease progression (Supplementary Table 5). Other patient characteristics and clinical parameters (i.e., age, GTV, re‐RT modality, smoking, and dissemination status) also demonstrated no significant association with PFS (i.e., local or disease progression at any localization; Supplementary Tables 5 and 6). When included in a multivariable Cox proportional hazard model adjusted for age, re‐RT modality, GTV, and dissemination status, residual ctDNA levels at the end of re‐RT did not reach the level of significance (Wald‐test, *p* = .075; HR = 5.74; 95% CI = 0.84–39.25; Supplementary Table 7), however, presented the highest predictive value of the included variables.

### 3.6 Decreasing ctDNA levels toward the end of re‐irradiation identified patients with short progression‐free survival

Next, we tested whether dynamic reductions in ctDNA levels were informative about duration of tumor control. To this end, we considered ctDNA changes in response to re‐RT (i.e., baseline vs. 5, 10 fractions or re‐RT end) and on‐treatment reductions (i.e., 5 fractions vs. 10 fractions, 5 fractions vs. re‐RT end and 10 fractions vs. re‐RT end). Relative ctDNA changes were calculated based on ctCPA scores or CPA scores without information from prior sampling time points (if ctCPA scores were not available for the given patient). No ctDNA detectability cut‐offs were applied for this analysis. We evaluated multiple ctDNA change thresholds to separate our patient cohort, ranging from +50% to −50% difference from the earlier to the later sampling time point (with 10% incremental steps). Solely, ctDNA reductions from re‐RT baseline and from the 5‐fraction time point to the end of re‐RT identified patient populations with significantly shorter PFS (Supplementary Table 6). Hereby, the CPA score decrease by ≥30% from 5 fractions to re‐RT end resulted in the most significant separation (log‐rank, *p* = .005; HR = 4.63; 95% CI = 1.42–15.11; Figure 3D). However, the ≥30% ctDNA reduction was no independent predictor for short PFS when included into a multivariable Cox regression model with age, re‐RT modality, GTV, and dissemination status (Wald‐test, *p* = .213; HR = 4.06; 95% CI = 0.447–36.96).

### 3.7 ctDNA levels showed no association with overall survival

We found no relation between OS and any of the previously investigated ctDNA measures as well as patient and clinical characteristics (Supplementary Table 6). These included the detectability of ctDNA at any pre‐ and on‐treatment time point and ctDNA level reductions in response to therapy.

### 3.8 ctDNA dynamics during and after re‐irradiation reflected therapy response and failure

**FIGURE 4:**
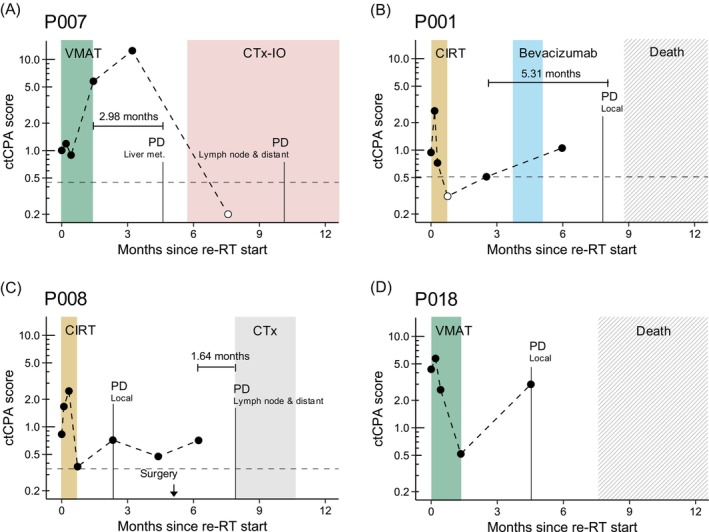
Representative patients (A–D) illustrating the utility of the ctCPA score for disease monitoring in HNC patients under re‐radiotherapy (re‐RT). Administered radio‐ and systemic therapies are represented by shaded areas. Time points of disease progression (PD) and PD location (if available) are denoted by vertical lines. Horizontal, dotted lines indicate the patient‐specific detectability threshold of the ctCPA score (i.e., maximum score across 57 healthy donors). Filled dots highlight samples with detectable ctCPA scores. Early signs of PD in months (i.e., increasing ctCPA scores prior to radiographic tumor progression) are highlighted in gray.

Recurrent locally advanced HNC patients often present with short disease‐free and overall survival.^1^, ^2^, ^3^, ^47^ Re‐irradiation can prolong survival of these patients compared to standard‐of‐care chemotherapy, however, time to disease progression and OS remains modest.^3^, ^9^ Longitudinally collected liquid biopsies facilitate real‐time disease monitoring^16^, ^17^, ^18^ and could provide early signs of tumor progression^14^, ^15^, ^16^ essential for disease management in this patient population. In this study, we evaluated the feasibility of disease monitoring using ctCPA scores as measure for chromosomal instability in the circulation, focusing on patients with at least one ctDNA‐positive sample (*n* = 8). ctCPA scores were calculated by integrating CNV information up to the re‐RT end time point. We found decreasing ctCPA scores from re‐RT baseline to the end of re‐RT in seven out of eight patients (Supplementary Figure 5A). Only patient P007 marked a ctCPA score increase which was in line with a subsequently diagnosed liver metastasis, not targeted by re‐RT (Figure 4A). Furthermore, ctCPA score increases between re‐RT end and the plasma specimen collected closest to disease progression (PD) were observed in seven out of eight patients (Supplementary Figure 5B). These observations highlighted the reflection of initial response to re‐RT and disease recurrence by chromosomal instability measured in circulation. Patients P001, P007, P008, and P018 (Figure 4A–D) exemplified cases that reflected radiographically determined disease kinetics both in response to re‐RT and subsequent lines of salvage treatments. For instance, rising ctCPA scores in patient P001 after termination of re‐RT—and despite administration of salvage immunotherapy—were accompanied by imminent PD and patient death (Figure 4B). In five patients (i.e., P001, P003, P006, P007, and P009), we observed rising ctCPA scores prior to radiographic tumor progression, potentially marking early signs of molecular PD (Figure 4 and Supplementary Figure 6). On average, PD detection by rising ctDNA levels in these patients preceded radiology by 3.30 months (range 1.34–5.31). Furthermore, increased ctDNA release during re‐RT—that is, ctCPA scores surpassing the baseline value at 5 or 10 fraction of re‐RT—was evident in five out of eight patients (P001, P006, P007, P008, and P018). We observed no association between these ctCPA score increases and the time point of blood sampling with respect to the application of RT.

## 4 DISCUSSION

Local recurrence is a common cause of treatment failure in advanced HNC patients and associated with poor survival.^1^, ^2^, ^3^, ^47^ Although re‐irradiation was demonstrated to result in favorable prognosis when compared to chemotherapy, second disease progression remains frequent and biomarkers predicting therapy success are urgently needed.^1^, ^2^, ^4^ This study systematically evaluated the association between ctDNA dynamics and clinical outcome in locally recurrent HNC patients under re‐irradiation.

We analyzed lcWGS data with the WisecondorX pipeline^36^ to assess genome‐wide CNVs in longitudinally collected plasma and used the extent of chromosomal instability per sample (CPA score) to indicate the presence of ctDNA. This approach is universally applicable since no prior knowledge of patient‐specific alterations or HPV status is required. Previous studies demonstrated the measurability of ctDNA in the circulation of HNC patients and reported detection rates between 30% and 100%, depending on the tumor stage and employed ctDNA assay.^16^, ^23^, ^25^, ^48^, ^49^ Locoregional recurrent disease, as observed in 15 out of 16 patients in this cohort, was shown to result in diminished ctDNA detectability when compared to metastatic disease progression (30% vs. 70% ctDNA‐positive patients detected).^48^ We found ctDNA in 50% of patients which reflected previous reports on ctDNA detectability in locally recurrent and non‐metastatic HNC patients by targeted mutation (30%,^48^) and lcWGS‐based CNV analysis (52%^25^). The validity of our ctDNA measure was further corroborated by the identification of CNVs commonly found in tumor tissue of HNC patients, such as the amplification of 3q and 8q as well as deletion of 3p, 9p, and 13q.^24^

To enhance CNV‐based ctDNA detection in our cohort, we introduced a methodology designed to use CNV information of longitudinal plasma specimens to increase assay sensitivity. Broadly, this approach (1) identifies CNVs in the current and all previous samples of one patient, (2) integrates this information into a cumulative CNV profile, and (3) quantifies chromosomal instability (ctCPA score) focusing on genomic regions known to be altered in the given patient. This concept follows the logic of tumor tissue‐informed ctDNA detection, wherein a tumor‐specific feature space is defined to increase the signal to noise ratio in WGS data.^46^ Ten additional ctDNA‐positive samples were detected by ctCPA scores, emphasizing the improved sensitivity without the requirement of additional sequencing efforts. Importantly, this strategy could be applied to enhance ctDNA detection in clinical settings where obtaining tissue biopsies is not feasible. Given that ctCPA scores were derived from patient‐specific CNVs, their significance lies in indicating ctDNA‐positivity and longitudinal disease assessment, rather than inter‐patient comparisons. Furthermore, the prerequisite of identifying at least one de novo CNV per patient precludes the detection of additional ctDNA‐positive patients.

An increasing body of evidence implies that on‐treatment ctDNA changes as well as residual ctDNA levels after therapy relate to clinical outcome.^18^, ^19^, ^20^, ^21^, ^50^ We found that residual ctDNA during re‐RT (i.e., after 5 and 10 fractions) and immediately after irradiation identified patients with short PFS. Furthermore, a decrease in ctDNA toward re‐RT termination (i.e., baseline vs. re‐RT end and 5 fractions vs. re‐RT end) was accompanied by early disease progression. In particular early response prediction could have significant clinical impact. For instance, residual ctDNA or ctDNA clearance after 5 or 10 fractions of re‐RT could guide subsequent radiation dosage (de‐)escalation to improve therapy success or spare toxic side‐effects. Moreover, detectable ctDNA levels after the last scheduled re‐RT administration could serve as indication for early initiation of subsequent therapy lines. In contrast to recent studies,^20^, ^21^, ^50^ we observed no prognostic value of baseline ctDNA levels. This might be explained by the heterogeneous pre‐treatment of the HNC patients of this study as well as the varying analytes and analytical methods used (i.e., mutations vs. CNVs and panel‐sequencing vs. lcWGS). Also, literature suggests a stronger correlation between ctDNA measures in response to therapy than baseline ctDNA levels.^50^ In the presented cohort, we found no association between ctDNA and OS in any of the investigated time points. A plausible explanation for this observation are the non‐uniform salvage therapies administered and their concomitant variable effect on long‐term survival. Through tracking of ctCPA scores in sequential plasma samples, we highlighted that ctDNA reflects tumor dynamics associated with response to re‐RT as well as successive salvage therapies. In 5 patients, we demonstrated that liquid biopsies can indicate disease progression in advance of clinical imaging. In the future, the identification of early signs of PD by ctDNA could aid clinical decision‐making, recommending patients at risk to narrow radiological surveillance or to early initiate systemic therapy in case of imminent progression. However, interventional studies are required to elucidate the potential benefits of ctDNA‐based therapy response prediction and disease monitoring.

In contrast to literature data,^43^, ^44^ we found no correlation between ctDNA levels and radiologically determined tumor burden at re‐RT baseline. We propose that this could be attributed to the extensive pre‐treatment of our cohort. A previous study showed in small cell lung cancer patients that the correlation between mutant allele frequencies in plasma and GTV increased when solely treatment naive patients are considered.^44^ Despite the lacking correlation at baseline, we observed elevated ctDNA levels in large tumors (i.e., exceeding the cohorts median GTV) when all plasma samples were considered. Hence, baseline GTV could identify patients more likely to benefit from ctDNA‐based therapy monitoring.

The main limitations of this study comprise its retrospective character, the small cohort size and the heterogeneous pre‐treatments as well as salvage therapies administered post re‐RT. Ideally, our findings should be confirmed in a larger prospective study. In addition, we detected no ctDNA in 8 out of 16 patients, including two patients (P004 and P014) with large tumors. The analysis of complementary biomarkers (e.g., mutations, DNA methylation^22^, ^25^) and/or cfDNA fragmentation features^13^, ^40^ as well as incorporating the collection of supplementary bio fluids (e.g., saliva or oral rinses^49^) should be evaluated in future studies to enhance ctDNA detectability and ensure that more patients can benefit from liquid biopsies.

## 5 CONCLUSIONS

To our knowledge, this is the first study to systematically assess the utility of CNV‐based ctDNA quantification as predictive biomarker in locally recurrent advanced HNC patients under re‐RT. We convincingly demonstrated that CNVs can be detected in plasma of HNC patients and devised a strategy to incorporate CNV profiles of longitudinal samples to enhance ctDNA detection. This approach could serve as a blueprint for sensitive CNV analysis in future studies. The defined plasma sampling intervals of this study allowed us to demonstrate the predictive value of residual ctDNA during and immediately after re‐RT and showcased the suitability of ctDNA for longitudinal therapy monitoring.

## AUTHOR CONTRIBUTIONS

**Florian Janke:** Conceptualization; data curation; formal analysis; methodology; visualization; writing – original draft; writing – review and editing. **Florian Stritzke:** Conceptualization; data curation; formal analysis; methodology; visualization; writing – original draft; writing – review and editing. **Katharina Dvornikovich:** Formal analysis; methodology; visualization; writing – review and editing. **Henrik Franke:** Formal analysis; methodology; visualization; writing – review and editing. **Arlou Kristina Angeles:** Formal analysis; methodology; visualization; writing – review and editing. **Anja Lisa Riediger:** Formal analysis; methodology; visualization; writing – review and editing. **Simon Ogrodnik:** Formal analysis; methodology; visualization; writing – review and editing. **Sabrina Gerhardt:** Formal analysis; methodology; visualization; writing – review and editing. **Sebastian Regnery:** Formal analysis; methodology; visualization; writing – review and editing. **Philipp Schröter:** Formal analysis; methodology; visualization; writing – review and editing. **Lukas Bauer:** Formal analysis; methodology; visualization; writing – review and editing. **Katharina Weusthof:** Formal analysis; methodology; visualization; writing – review and editing. **Magdalena Görtz:** Formal analysis; methodology; visualization; writing – review and editing. **Semi Harrabi:** Formal analysis; methodology; visualization; writing – review and editing. **Klaus Herfarth:** Formal analysis; methodology; visualization; writing – review and editing. **Christian Neelsen:** Formal analysis; methodology; visualization; writing – review and editing. **Daniel Paech:** Formal analysis; methodology; visualization; writing – review and editing. **Heinz‐Peter Schlemmer:** Formal analysis; methodology; visualization; writing – review and editing. **Amir Abdollahi:** Formal analysis; methodology; visualization; writing – review and editing. **Sebastian Adeberg:** Conceptualization; formal analysis; methodology; visualization; writing – original draft; writing – review and editing. **Jürgen Debus:** Conceptualization; formal analysis; methodology; supervision; visualization; writing – original draft; writing – review and editing. **Holger Sültmann:** Conceptualization; formal analysis; funding acquisition; methodology; project administration; supervision; visualization; writing – original draft; writing – review and editing. **Thomas Held:** Conceptualization; formal analysis; funding acquisition; methodology; project administration; supervision; visualization; writing – original draft; writing – review and editing.

## FUNDING INFORMATION

This work was supported by the Deutsche Krebshilfe. The funder had no role in the design of the study; in the collection, analyses, or interpretation of data; in the writing of the manuscript, or in the decision to publish the results.

## CONFLICT OF INTEREST STATEMENT

TH reports advisory board fees from Merck and Sanofi outside the submitted work; JD reports grants from Accuray Inc., grants from RaySearch Laboratories AB, grants from Vision RT limited, grants from Merck Serono GmbH, grants from Siemens Healthcare GmbH, and grants from PTW‐Freiburg Dr. Pychlau GmbH, outside the submitted work. SA reports participations on advisory boards of Accuracy Inc., Sanofi Genzyme, Novartis GmbH as well as grants from Novocure GmbH, Accuracy Inc., AstraZeneca GmbH, Bristol Myers Squibb & Co., MSD, Merck KGaA, Fakultät Heidelberg, Sanofi (all outside the submitted work). The other authors declare no conflict of interest.

## ETHICS STATEMENT

This study was approved by the ethical committee of Heidelberg University (S‐130/2021 and S‐708/2018) and written, informed consent was provided by all study participants. This study was performed in accordance with the Declaration of Helsinki. Patients were recruited as part of the CARE phase II clinical trial (NCT04185974).

## Supporting information


DATA S1:

DATA S2:

## Data Availability

Plasma whole genome sequencing data that support the findings of this study are deposited in the European Genome‐Phenome Archive (EGA) under accession number EGAS50000000163. Sequencing data from Peneder et al. (https://doi.org/10.1038/s41467-021-23445-w) was previously deposited to the EGA under accession EGAS00001005127. All source code is publicly available on GitHub (https://github.com/jankef/ctDNA‐predicts‐outcome‐in‐head‐and‐neck‐cancer). Further information is available from the corresponding author upon request.
