# Supplementary material for: Early circulating tumor DNA changes predict outcomes in head and neck cancer patients under re‐radiotherapy
Source: Int J Cancer. 2024 Aug 30;156(4):853–64. doi: 10.1002/ijc.35152 (PMC11661516; doi:10.1002/ijc.35152)
Supplement: Supplementary file 1 — DATA S1: [file IJC-156-853-s001.pdf]

Early circulating tumour DNA changes predict outcomes in head and neck cancer patients under re-radiotherapy

Florian Janke*, Florian Stritzke*, Katharina Dvornikovich, Henrik Franke, Arlou Kristina Angeles, Anja Lisa Riediger, Simon Ogrodnik, Sabrina Gerhardt, Sebastian Regnery, Philipp Schröter, Lukas Bauer, Katharina Weusthof, Magdalena Görtz, Semi Harrabi, Klaus Herfarth, Christian Neelsen, Daniel Paech, Heinz-Peter Schlemmer, Amir Abdollahi, Sebastian Adeberg, Jürgen Debus, Holger Sültmann†, Thomas Held†

* These authors contributed equally to this work

† These authors contributed equally to this work and share senior authorship

Table of contents

Supplementary Fig. 1 3
Supplementary Fig. 2 4
Supplementary Fig. 3 5
Supplementary Fig. 4 6
Supplementary Fig. 5 7
Supplementary Fig. 6 8
Supplementary Tab. 1 9
Supplementary Tab. 2 9
Supplementary Tab. 3 9
Supplementary Tab. 4 9
Supplementary Tab. 5 9
Supplementary Tab. 6 9
Supplementary Tab. 7 9

**Note: All supplementary tables are provided in a separate excel document.*

Supplementary Fig. 1

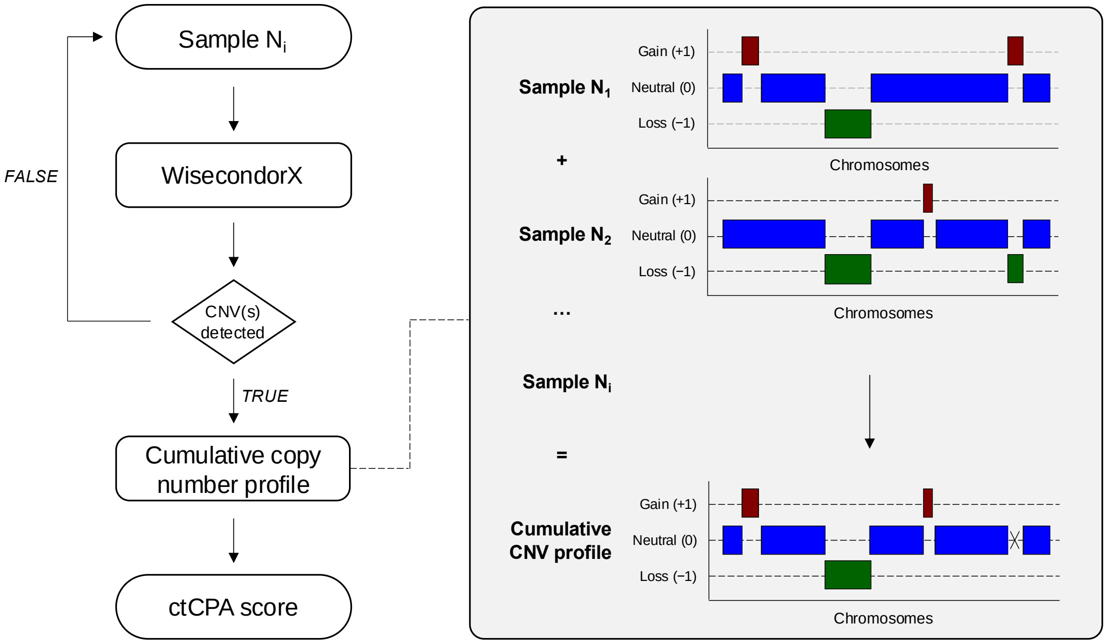

Supplementary Fig. 1: ctDNA-informed CPA score (ctCPA) determination workflow. The flowchart (left) illustrates the ctCPA score calculation workflow for Patient N with i representing individual samples of this patient. WisecondorX is used to create copy number profiles. If CNVs are detected in the given sample, these are added to a cumulative, patient-specific copy number profile. The cumulative copy number profile (right) combines CNVs detected in the current and all previous samples of patient N. Next, the ctCPA score is calculated by weighting the z-scores of individual segments according to their copy number state in the cumulative profile, followed by their summation. CNV, copy number variation; CPA, copy number profile abnormality.

Supplementary Fig. 2

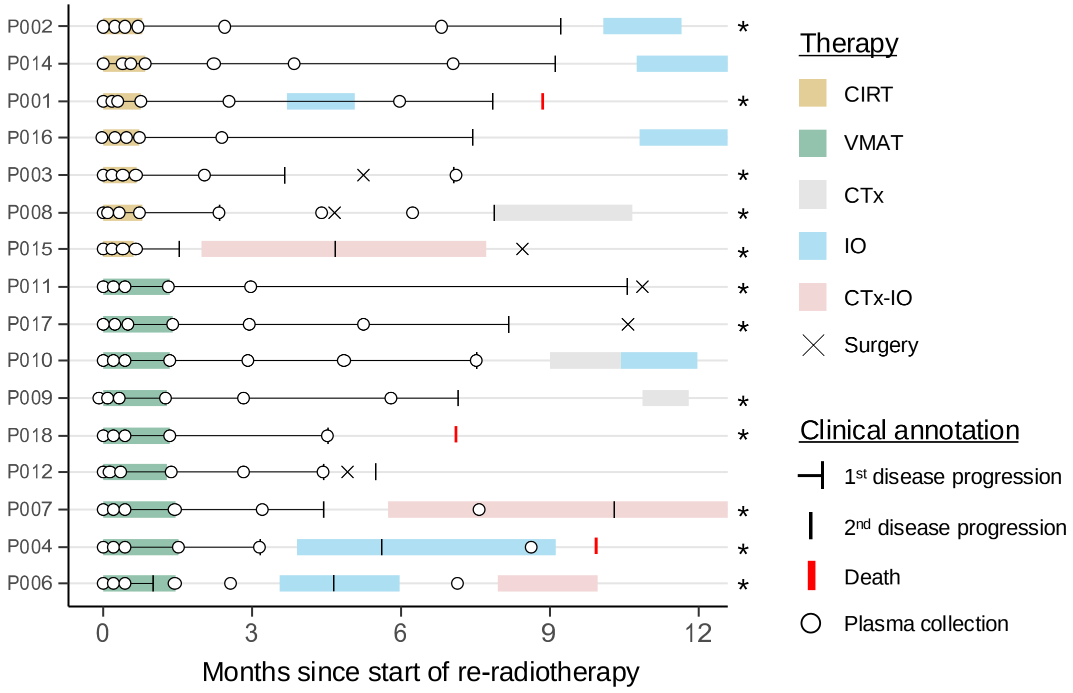

Supplementary Fig. 2: Swimmer plot illustrating plasma collection time points, administered therapy regimes as well as time of disease progression during the first 12 months after initiation of re-radiotherapy. Patients are ordered by re-radiotherapy type and duration of progression-free survival. Asterisks indicate patients that deceased during or after the illustrated 12 months follow-up period. CIRT, carbon ion radiotherapy; CTx, chemotherapy; CTx-IO; chemo-immunotherapy combination; VMAT, volumetric modulated arc therapy.

Supplementary Fig. 3

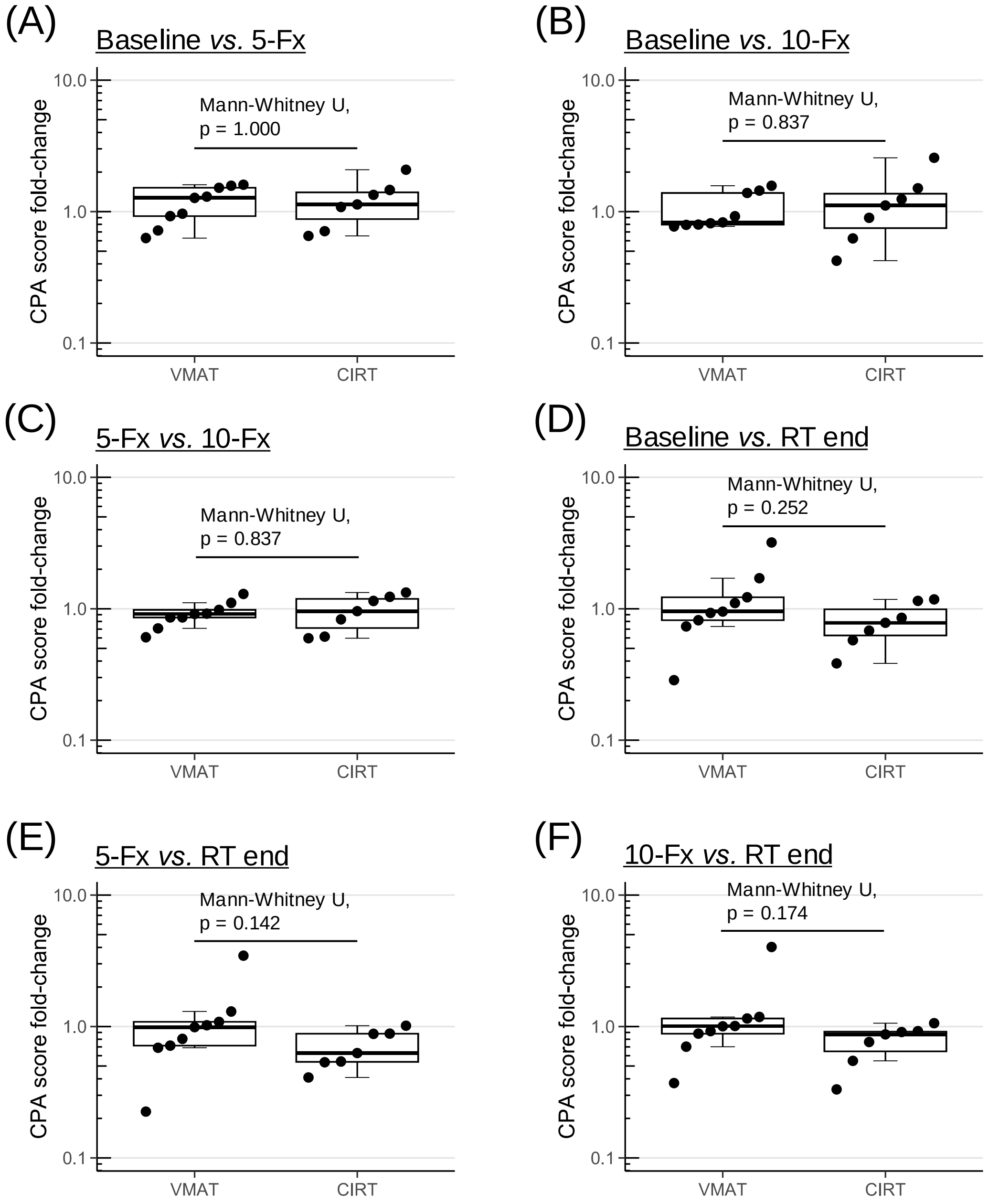

Supplementary Fig. 3: CPA score change from baseline to 5 re-RT fractions (5-Fx; A), baseline to 10-Fx (B), 5-Fx to 10-Fx (C), baseline to the end of re-RT (D), 5-Fx to re-RT end (E), and 10-Fx to re-RT end. CPA score changes were compared between volumetric modulated arc therapy (VMAT) and carbon ion radiotherapy (CIRT). Box plots represent median, upper and lower quartile with Tukey Whiskers.

Supplementary Fig. 4

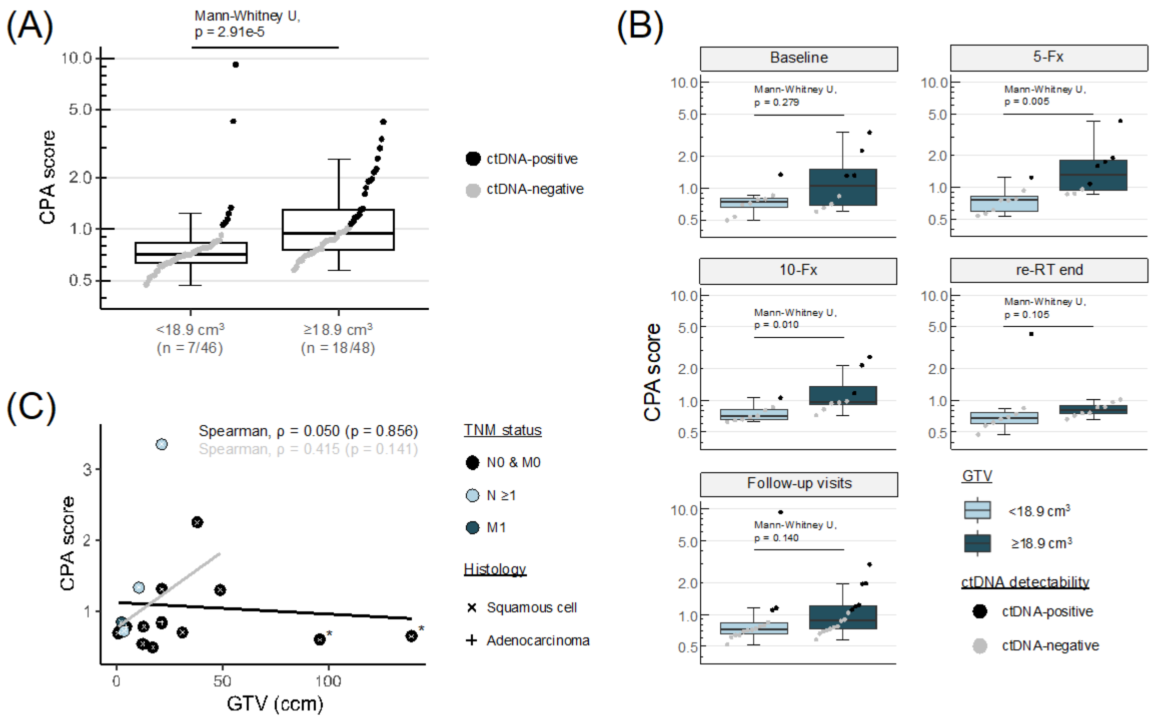

Supplementary Fig. 4: (A) Comparison of CPA scores in head and neck cancer patients stratified by the cohort´s median GTV (18.9 cm^3). ctDNA detectability is color-coded in black (positive) and gray (negative), according to the detectability threshold (dashed line; maximum CPA score in healthy donor cohort). ctDNA-positive and total sample number per group is given in parentheses. (B) CPA score comparison between by GTV of individual sampling time points (i.e., at baseline, after 5, 10 fractions and at the end of re-RT as well as at follow-up visits). Box plots represent median, upper and lower quartile with Tukey Whiskers and statistical significance between groups was assessed by Mann-Whitney U tests. (C) Correlation between baseline CPA scores and GTVs. Linear regression line and corresponding Spearman correlation coefficients are given in the plot (black including and gray excluding outliers). Asterisks mark outliers removed for the linear regression shown in gray. Patient histology and information on disease dissemination (by TNM annotations) is highlighted.

Supplementary Fig. 5

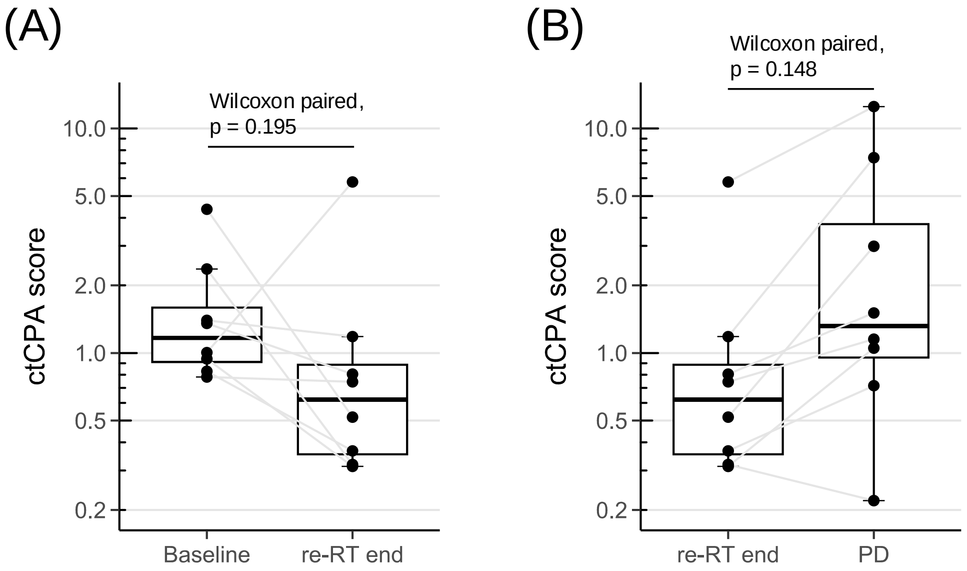

Supplementary Fig. 5: Per patient ctCPA score dynamics from baseline to the end of re-radiotherapy (re-RT; A) and from re-RT end to the plasma sample collected closest to disease progression (PD; B). For patient P006, the second PD time point was considered as the first PD occurred already during re-RT. Statistical significance between groups was assessed by Wilcoxon´s paired test. Box plots represent median, upper and lower quartile with Tukey Whiskers. CPA, copy number profile abnormality.

Supplementary Fig. 6

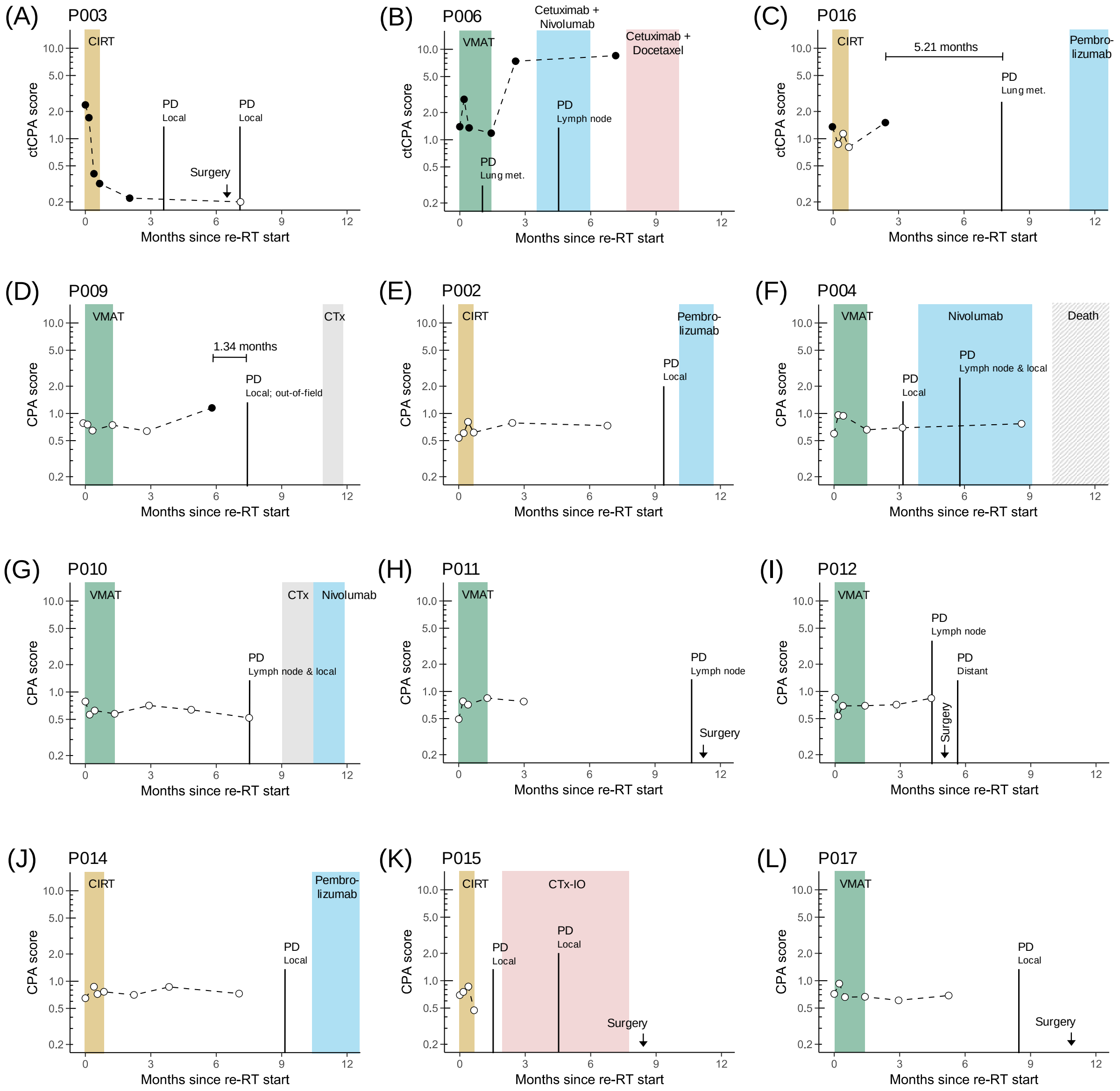

Supplementary Fig. 6: Remaining (ct)CPA score kinetics during and after re-radiotherapy (re-RT). Disease kinetics are represented by ctCPA scores (if available; A-C) or CPA scores (D-L). Therapies are displayed by shaded areas (re-RT and systemic therapies) and arrows (surgery). Disease progression (PD) time points (plus PD locations) are given by vertical lines. CtDNA detectability thresholds are given by the dotted lines and ctDNA-positive and –negative samples are highlighted via black and white fillings, respectively. Lead time (in months) is shown by gray, horizontal bars.

Supplementary Tab. 1

Demograhic and clinical information per patient.

Supplementary Tab. 2

cfDNA and sequencing statistics per sample

Supplementary Tab. 3

Summary of CPA score comparisons between cases and controls at different sampling time points. ctDNA-positive and total number of samples per group are given in column ‘#Samples’. CPA, copy number abnormality; FU, follow-up; Fx, fraction; RT, radiotherapy.

Supplementary Tab. 4

Summary of CPA score differences between demographic and clinical patient characteristics. Comparisons are shown for all longitudinal samples and for each sampling time point separately. The ctDNA-positive and the total number of samples per group are given in column ‘#Samples’. Dissemination refers to TNM status at re-irradiation baseline. ‘Distant’ includes patients with N≥1 and/or M1. GTV was determined at re-radiotherapy baseline. Age and GTV separation was performed according to the cohort median. CIRT, carbon ion radiotherapy; ctDNA, circulating tumour DNA; adenocarcinoma; CPA, copy number abnormality; GTV, gross tumour volume; RT, radiotherapy; SCC, squamous cell carcinoma; TNM, tumour-node-metastasis; VMAT, volumetric modulated arc therapy.

Supplementary Tab. 5

Univariate survival analysis comparing local progression-free survival according to ctDNA detectability and patient characteristics. P-values <0.05 are given in bold letters.

Supplementary Tab. 6

Univariate survival analysis comparing progression-free and overall survival according to ctDNA detectability, relative ctDNA changes and patient characteristics. For PFS assessment, disease progression at any site (i.e., local, regional or distant) was considered. For relative ctDNA changes results are depicted for comparisons with a minimum group size of 3 in either group. P-values <0.05 are given in bold letters.

Supplementary Tab. 7

Multivariable Cox regression analysis including age, re-RT modality, GTV, dissemination status and ctDNA-positivity at the end of re-RT. Only parameters available for all 16 patients were

included in this analysis. For numeric parameters, patients were dichotomized based on the cohort´s median value. P-values <0.05 are given in bold letters.
